# Oculus: faster sequence alignment by streaming read compression

**DOI:** 10.1186/1471-2105-13-297

**Published:** 2012-11-13

**Authors:** Brendan A Veeneman, Matthew K Iyer, Arul M Chinnaiyan

**Affiliations:** 1 Department of Computational Medicine and Bioinformatics, University of Michigan Medical School, Ann Arbor, MI, 48109, USA; 2 Michigan Center for Translational Pathology, University of Michigan Medical School, Ann Arbor, MI, 48109, USA; 3 Department of Pathology, University of Michigan Medical School, Ann Arbor, MI, 48109, USA; 4 Howard Hughes Medical Institute, University of Michigan Medical School, Ann Arbor, MI, 48109, USA; 5 Department of Urology, University of Michigan Medical School, Ann Arbor, MI, 48109, USA

**Keywords:** DNA nucleotide sequence alignment streaming identity redundancy compression software algorithm

## Abstract

**Background:**

Despite significant advancement in alignment algorithms, the exponential growth of nucleotide sequencing throughput threatens to outpace bioinformatic analysis. Computation may become the bottleneck of genome analysis if growing alignment costs are not mitigated by further improvement in algorithms. Much gain has been gleaned from indexing and compressing alignment databases, but many widely used alignment tools process input reads sequentially and are oblivious to any underlying redundancy in the reads themselves.

**Results:**

Here we present Oculus, a software package that attaches to standard aligners and exploits read redundancy by performing streaming compression, alignment, and decompression of input sequences. This nearly lossless process (> 99.9%) led to alignment speedups of up to 270% across a variety of data sets, while requiring a modest amount of memory. We expect that streaming read compressors such as Oculus could become a standard addition to existing RNA-Seq and ChIP-Seq alignment pipelines, and potentially other applications in the future as throughput increases.

**Conclusions:**

Oculus efficiently condenses redundant input reads and wraps existing aligners to provide nearly identical SAM output in a fraction of the aligner runtime. It includes a number of useful features, such as tunable performance and fidelity options, compatibility with FASTA or FASTQ files, and adherence to the SAM format. The platform-independent C++ source code is freely available online, at http://code.google.com/p/oculus-bio.

## Background

Nucleic acid sequencing throughput has grown exponentially for the past ten years, and is expected to continue to shatter Moore’s law [1]. Though the highly anticipated onslaught of inexpensive sequencing empowers exciting new biological studies, it also presents a critical problem: the skyrocketing computational costs of sequence analysis [2]. Computers may become the bottleneck of genomics research if these growing processing demands are not mitigated by improvements in software algorithms, especially in light of the sequencing demands of personalized medicine.

Much intellectual effort has been invested in minimizing the time required to align a single read against an indexed database. When performed sequentially, each sequence in the input is processed individually, such that the sum of the alignment times of the input sequences is the total running time. Today’s fastest and most widely used aligners, such as Bowtie [3], BWA [4], MAQ [5], RazerS [6], and BLAST [7], process input reads sequentially. These aligners can typically be configured to be consistent and guarantee that identical copies of an input sequence will produce identical alignment results. Therefore, given a set of input reads with ample redundancy, we envisioned that alignment time could be reduced without compromising accuracy by distilling the unique set of sequences and aligning them using a sequential alignment tool.

Harnessing redundancy in sequence alignment input is not a new concept. BLAST + gains a performance benefit by saving alignments within batches [8]. Cloudburst and CloudAligner use MapReduce, and feature a shuffle step wherein seed sequences in the query and database are brought together and combined [9,10]. SEAL also uses MapReduce; it effectively parallelizes BWA, and can remove duplicate reads by comparing alignment position, after aligning all of them [11]. Similarly, SlideSort sorts together sequences with common substrings [12], and mrsFast uses a sophisticated blocking map to identify unique seeds before performing a direct map-to-map comparison [13]. Finally, Fulcrum performs hashing on seed sequences using MapReduce to conserve computation time in genome assembly [14]. While all of these are excellent tools in their own application spaces, sequential aligners such as Bowtie and BWA enjoy extensive support, remain popular for many applications, and can benefit from the same approach. Furthermore, decoupling the process of compressing input reads from the alignment kernel itself could be productive, as improvements to both algorithms can proceed independently. To date, no application exists that performs streaming read compression in a generalized way.

## Methods

We explored the nature of read redundancy across thirteen publicly available next-generation nucleotide sequencing datasets. In a series of experiments we measured the contributions of the application (whole genome, targeted exome capture, RNA-Seq, and ChIP-Seq), read length, and sequencing depth to overall read redundancy, measured in the percentage of unique reads. Using these observations, we wrote the streaming read compression algorithm Oculus and constructed a model to determine the value of streaming read compression for a given dataset. Finally, we benchmarked Oculus on full sequencing datasets.

### Sequence data profiling

We evaluated thirteen publicly available datasets that were representative of the major applications of high-throughput sequencing, identified here by their NCBI Sequence Read Archive (SRA) accession numbers. There were five RNA-Seq datasets (ERS025093 (pooled), and SRR097790, SRR097792, SRR097786, and SRR097787 from the iDEA challenge), three genome datasets (SRR097850 and SRR097852, also from the iDEA challenge, and ERR000589), three Exome sequencing datasets (SRR098490, SRR098492, and SRR171306), and finally two ChIP-Seq datasets: (SRR227346, and SRR299316 + SRR299313 (pooled)). The ChIP-Seq data was downloaded from the ENCODE Project [15], hosted on the UCSC genome browser. Illumina, Inc. carried out the IDEA dataset sequencing, first used by Sun et al. [16]. Additional run metadata can be found in Additional file 1: Table S1.

### Sequencing type

**Figure 1:**
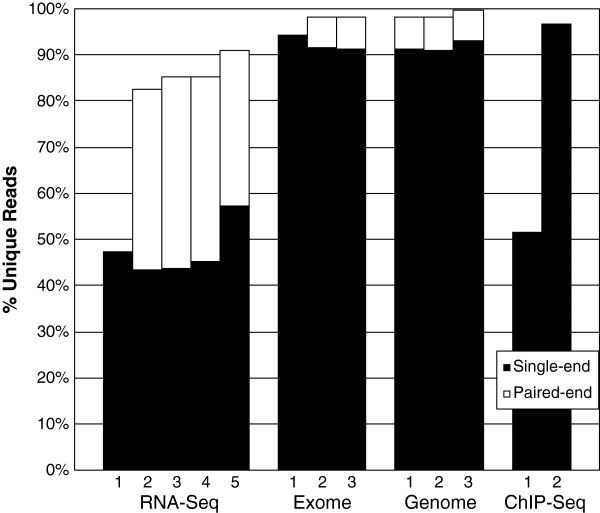
**RNA-Seq compresses better than other sequencing platforms.** Each benchmark dataset was randomly subset to the lowest common denominator number of reads (24 million) and read length (36 bases). Subsequently, Oculus computed the unique read fraction for each dataset using the reverse-complement option. For data with paired-ends available, 12 million pairs were used to computer %unique reads. RNA-Seq #1, Exome #1, and ChIP-Seq #1-2 did not have available paired-end data.

The sequencing datasets we selected varied widely in their composition. We compared read redundancy between sequencing types by standardizing the number of reads per dataset to 24 million with random subsetting, and read length to 36 bases with 3’ end trimming (both lowest common denominators). RNA-Seq had relatively redundant reads; only 43% to 57% of each single-end dataset was unique (Figure 1). In contrast, Exome and Genome sequencing had very little read redundancy. The two ChIP-Seq datasets had disparate content, varying greatly in their % unique reads – without delving into the specifics of those samples, we believe this may reflect the wide variety of ChIP-Seq applications. As expected, paired-end data compressed less well than single-end, since paired-end compression requires identity on both reads.

### Depth of coverage and read length

**Figure 2:**
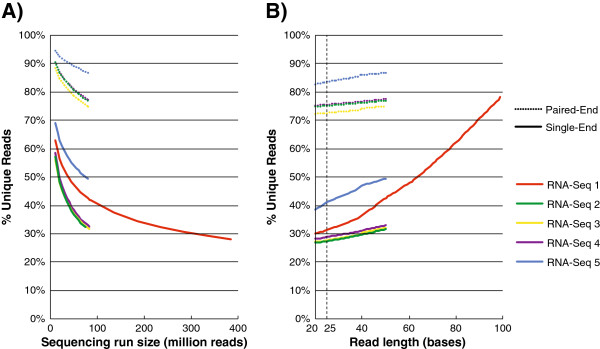
**Compression improves for larger sequencing runs and shorter read lengths.A**) Each RNA-Seq dataset was trimmed to 50-base reads, and %unique reads was computed for a series of simulated sequencing run sizes (between 10 million single-end or paired-end reads and their original size). **B**) Each RNA-Seq dataset was randomly subset to 79 million single-end or paired-end reads, and %unique reads was computed for a series of simulated read lengths by trimming from the end (between 20 bases and their original read size). 25 bases is a typical sequence length that advanced RNA-Seq pipelines such as TopHat may use for segmented alignment.

Given some fixed input DNA from which fragments are sampled, each incremental read will be more likely to duplicate previous reads. In particular, RNA-Seq reads may disproportionately reflect highly expressed genes, suggesting that higher sequencing coverage could have a nonlinear effect on read redundancy [17]. Therefore, we measured the impact of coverage depth/sequencing run size (number of reads) and read length on the unique read percentage of each dataset, treating reads individually (single-end) or as pairs (paired-end) (Figure 2). We fixed the read length for RNA-Seq runs and evaluated % unique reads for a series of random fractions of the original datasets. As predicted, larger sequencing runs corresponded logarithmically to a lower unique fraction of the datasets (Figure 2A). The unique read fraction varied between 56-69% for 10 million reads, 32-49% for 25 million reads, and 28% for 385 million reads in RNA-Seq dataset #1. The differences between datasets likely relates to sample biology and preparation. Next, we fixed coverage depth and evaluated the percentage of unique reads for a series of read lengths (trimming from the end) (Figure 2B). The impact of read length on uniqueness appeared to be exponential in one case (RNA-Seq #1, for which 100 bp reads were available) and linear in the rest (RNA-Seq #2-5). It’s interesting to note that some RNA-Seq algorithms, such as TopHat [18], dice unmapped reads into segments and align each piece individually. This might entail a ~3-fold alignment speedup for RNA-Seq dataset #1 by use of 25 base segments, if further communication between a streaming read compressor such as Oculus and Tophat’s core algorithm could be engineered.

## Implementation

**Figure 3:**
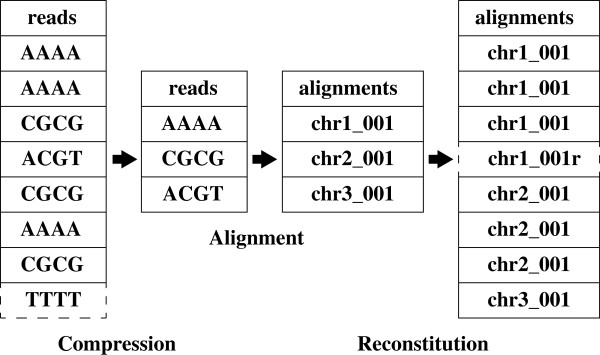
**Flowchart depicting Oculus behavior with example sequences.** As input is parsed, new sequences are passed into the aligner in the order they are observed. The aligner then performs normally, mapping each passed read to the database. Downstream of the aligner, Oculus expands the alignment file to reflect the count of each input sequence. Since compression and reconstitution are faster than alignment, there is a net reduction in runtime. In reverse-complement mode (Section 2.4), Oculus would remove the read sequence TTTT, having already seen AAAA, and print an additional alignment: chr-001 with reversed orientation. By default, Oculus treats AAAA and TTTT as distinct sequences – both would be passed into the aligner.

The overall architecture of Oculus is shown in Figure 3. Oculus reads FASTA or FASTQ input files, processes sequences into a compressed form, and compares them to a map containing all sequences it has seen before; new sequences are passed into the aligner as FASTA, while previously observed sequences increment counts in the map. At the reconstitution step, sequences in the SAM output file are then compared back against the map and re-printed as many times as they appeared in the input, correcting for alignment orientation. Paired-end sequences are handled by concatenating the two sequences to ensure the pair is unique. Oculus can wrap any aligner capable of producing SAM-formatted output.

By design, Oculus sacrifices FASTQ quality scores, read names beyond the first instance of the sequence, and the original order of the reads in the output. Optionally, users can direct Oculus to restore the original read names and quality scores by writing them to an intermediate file, sorting it, and reattaching them during the reconstitution step. This option incurs additional memory overhead, and additional time to sort the intermediate file.

### Data structures

Oculus uses hashmap data structures to store sequences in memory. Users can either compile in standard library (STL) hashmaps, or Google-SparseHash maps, which are faster and require significantly less memory (2 bits of overhead per entry) [19].

Optionally, users can direct Oculus at runtime to store unique reads in a separate hashset, reducing the burden on the hashmap to only redundant sequences. The effect of this is to reduce lookup times in the reconstitution step and total memory consumption, at the cost of more operations in the compression step. Hashsets are expected to be beneficial for lower redundancy input.

Oculus uses a modified version of MurmurHash2 to hash binary sequence data [20]. It has a low incidence of collision for binary data, and was recommended for use with Google-SparseHash by its developer (C. Silverstein, personal communication). To reduce collisions, the hash algorithm operates only on the sequence field of the compressed sequence objects.

### Binary compression

**Figure 4:**
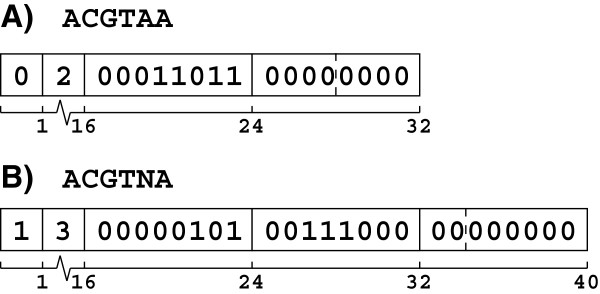
**Compressed sequence object (cseq) diagrams.** Numbers below the data fields indicate the 0-based index in bits from the left end. (**A**) The sequence ACGTAA contains no N’s, so its encoding bit is 0, indicating 2 bits per base. By that encoding, two bytes are required to store 6 nucleotides, so the size field is 2. The sequence field is populated by A = 00, C = 01, G = 10, T = 11, etc., with the right-most byte padded on the right by zeros. (**B**) Compression proceeds as before, until the N nucleotide is encountered, at which point the compression starts over and sets the encoding to 1, indicating 3 bits per base. At that compression, now 3 bytes are required to store 6 nucleotides, and the size field is updated accordingly. The sequence field is populated by A = 000, C = 001, G = 010, T = 011, N = 100, etc., and again the right-end is padded with 0 s.

Instead of storing sequences in memory as ASCII characters, Oculus uses compressed sequence objects of our own design (cseqs) (Figure 4). DNA sequences are dynamically compressed into 2 or 3 bits per base, depending on the presence of N nucleotides. Optionally, a 2-bit encoding can be forced if the user wishes for N’s to be evaluated as A’s. Each cseq has three fields: a representation bit indicating the nucleotide encoding, its size in memory, and a variable-length compressed sequence. Storing the size is necessary because null-termination is obviated by the possibility of null bytes in the sequence field.

The most obvious benefit of using cseqs is an approximate four-fold reduction in memory use. However, two engineering benefits also arise for cseq string comparison, which help efficiently resolve map collisions. Sequences with different lengths or representations can be differentiated by comparing the first byte in constant time (very quickly). Moreover, by comparing nucleotides in blocks instead of individually, comparison time is reduced four-fold. Memory for sequences is allocated in large chunks (default: 10kB), which reduces overhead greatly.

### Reverse complements

Lastly, Oculus can be directed to compress together reverse complements in single-end data, or reversed read order in forward-reverse oriented paired-end data, under the presumption that they should align to the same place in the database. This improves compression and therefore reduces aligner runtime. Using reverse complements is optional because BWA and Bowtie both use left-end seed sequences, so the orientation of the read can affect its alignment (though typically in a tiny fraction of sequences).

### Runtime model

We developed a model to predict the effectiveness of Oculus for any given data set. Given N_i_ input reads that compress to N_c_ sequences, and assuming s_a_ and s_o_ are the speeds of the aligner and Oculus, in reads/unit time, the following equations give the expected benefit of using Oculus as a fraction of the aligner’s run time.

(1) $$\begin{array}{ll} \text{Aligner Run Time}= & N_{i}{\mathrm{/s}}_{a}\\ \text{Oculus Run Time}= & \left(N_{i}{\mathrm{/s}}_{o}\right)+\left(N_{c}{\mathrm{/s}}_{a}\right)\\ \text{Run Time Ratio}=\left(\text{Oculus}/\text{Aligner}\right)= & \left(s_{a}{\mathrm{/s}}_{o}\right)+\left(N_{c}{\mathrm{/N}}_{i}\right) \end{array}$$

The aligner’s run time is simply the total number of input reads divided by the average alignment speed in reads per unit time of the aligner. In the second case, since Oculus passes some fraction N_c_ of N_a_ into the aligner, the aligner only has to do N_c_/s_a_ work. However, there’s also an overhead for Oculus on the order of the total number of input reads. The fractional benefit of using Oculus is therefore related only to the compression achieved and Oculus’s speed relative to the aligner it’s wrapping. We therefore derived processing rates in reads per second for Oculus and each aligner, for both single-end and paired-end data, using experimental results for the 50 and 51-mer datasets. Table 1 indicates the calculated ratio of the speed of the aligners to Oculus. Based on these parameters we predict that Oculus will have a runtime benefit for sequence data with greater than 10% redundant reads, and that benefits would scale linearly with the unique read fraction. This model discounts non-linear factors such as hash collisions, read length, percent successful alignment, and potentially, alignment location, and disk I/O will produce noise, but it is an effective rule of thumb.

**Table 1:** Relative processing speeds of Bowtie and BWA to Oculus, for single-end and paired-end data

		**SE**	**PE**
s_a_/s_o_	Bowtie	0.079	0.023
	BWA	0.017	0.015

s_a_ is the aligner’s speed, and s_o_ is Oculus's speed. Since speeds are measured in reads aligned per second, these values indicate that Oculus runs faster than the aligners, and relatively more fast for paired-end data than single-end data. As expected, Bowtie was measured to be faster than BWA, particularly for single-end data. Both aligners were run with default options.

### Benchmarking

We compared the performance of Oculus with BWA (version 0.5.9-r16) and Bowtie 1 (version 0.12.7 64-bit) by themselves. All alignment was performed against the reference human genome GRCh37/hg19.

Every benchmarking test was run on the Flux supercomputing cluster maintained by the Center for Advanced Computing at the University of Michigan, using single CPU cores of 2.67 GHz Intel X5650 processors, with 64 GB of 1333 MHz DDR3 memory, and distributed access disks. To reduce noise in runtime measurement from disk I/O, each benchmark test was run three times, and the average runtime is presented here. Memory consumption was much less noisy, so similar averaging was unnecessary in reporting memory use. Both aligners ran with entirely default options, and Oculus used only the reverse complement storage option, “--rc”.

To test consistency, we ran Bowtie using “-m 1” to eliminate multi-mapping reads, for which Bowtie reports one random alignment by default. We extracted alignment positions, sorted by read sequence (grouping together forward and reverse orientations), and counted and classified alignment differences. BWA has no such mono-mapping option, so we did not test Oculus’s wrapping of BWA for consistency (BWA was still tested for performance).

## Results

### Compression and performance

**Figure 5:**
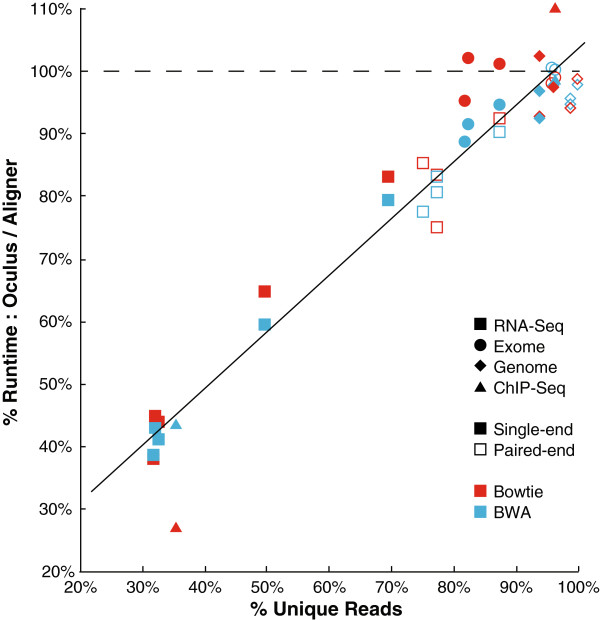
**Oculus provides a speedup that correlates linearly with % unique reads.** % Runtime represents the ratio of the runtime of Oculus, wrapping each aligner, to the runtime of the aligner by itself (in CPU time). To best demonstrate fractional benefit, Bowtie and BWA results are combined in this graph – individual run data is available in Additional file 1: Table S1. Oculus provided a speed benefit for points below the dashed line. These datasets span a variety of sequencing types, read number, and read length, which we hypothesized all contribute to the % unique reads for a sequencing run. See Additional file 1: Table S1 for individual sequencing run characteristics such as read number and read length.

Oculus yielded performance benefits that strongly correlated with the unique read fraction of each dataset (Figure 5). Notably, the single-end RNA-Seq datasets aligned in 49.7% as much time on average, i.e., they ran 2.0 times as fast in Oculus compared with Bowtie and BWA. The paired-end datasets compressed less well than their single-end counterparts; on average, the paired-end RNA-Seq datasets aligned 1.2x as fast. ChIP-Seq dataset #1 received the greatest performance benefit: its single-end Bowtie alignment ran 3.7x as fast. However, our Genome and Exome datasets, and ChIP-Seq dataset #2, were generally non-redundant and Oculus did not greatly outperform either aligner. This was consistent with our expectations - if reads are not redundant, they cannot be compressed, and the aligner will receive nearly the complete set of input reads. Since compressing and decompressing incurs a small time overhead, it follows that a nearly completely unique dataset might run more slowly.

Though BWA was much slower than Bowtie for single-end data, and somewhat slower for paired-end data, Oculus produced similar fractional speed improvements for the two aligners. Additionally, for the datasets tested, Oculus’s hashset option did not yield a significant improvement. For sequencing run information and exact CPU run times, see Additional file 1: Table S1.

### Consistency

Oculus maintained high fidelity to original alignments for every dataset. Defining accuracy as the percentage of input reads that Oculus mapped to exactly the same location as the aligners, on average Oculus was >99.9% accurate, and in the worst case was 99.874% accurate. For individual dataset accuracy, see Additional file 1: Table S1.

Since they change the seed sequence used in alignment, the vast majority of the differences (inaccuracies) produced were for reads that Oculus either reversed the orientation of (88% of single-end differences), or order of (67% of paired-end differences). Mostly these were previously unaligned reads that aligned and vice versa, but in some cases, an unambiguously mapped read actually changed alignment positions (single-end, 0.09% of differences; paired-end, 10.15% of differences). Though initially surprising, this can be explained by mismatches in seed sequences. Bowtie is less permissive of mismatches in the seed than at the end of a read under the assumption that read quality tends to be better toward 5’ end. Of two closely homologous regions of the genome, one may count as the best hit in the forward orientation, and the other in reverse orientation. For example:

CAGT - read

CATT – genome position 1

CCGT – genome position 2

In this case, if CA is the seed, position 1 would be the optimal alignment and the third base would count as a G-T mismatch. However, if the reverse-complement were aligned, and the seed proceeded from the opposite direction, position 2 would be optimal and the third base would be recorded as a C-A mismatch.

### Memory use

Oculus very consistently used (sequence length/4) + 20 bytes of memory per map entry. This 20-byte overhead comes from the forward and reverse count integers (4 each), the hash of the sequence (4), a pointer to the sequence (up to 8 on a 64-bit OS), the size field (2), and some heap memory structure overhead. Although these sum to 22 bytes, hash values are not stored multiple times for hash collisions, and pointer memory use varies by OS architecture, often using less than 8. This 20-byte overhead is halved for paired-end map entries, because each pair is stored together. Using the hashset option reduced memory use by about a third, by mitigating some of this overhead for unique reads.

Total memory use is therefore highly dependent on the quantity and redundancy of input sequence, but in a worst-case scenario (perfect non-redundancy), 100 million single-end 80mers will use about 3.7 GB of memory, on top of memory used by the aligner’s database. Redundancy translates linearly to reduction in memory use – if only half of those reads were unique, 1.85 GB would be required instead.

## Discussion

Our benchmarking tests suggest Oculus will generally perform very well with RNA-Seq data and on a case-by-case basis in other applications, particularly those with low complexity libraries. The likely source of benefit to RNA-Seq arises from highly expressed genes that are sequenced at great depth and generate multitudes of duplicate reads.

Shorter read length and larger datasets both correlated with higher redundancy in sequencing runs. The hidden variable of actual biological redundancy remains at large (particularly, the effects of PCR and the targeted scope of sequencing), but those two metrics provide good insight into the expected value of streaming read compression for a given sequencing application. We noted the added value Oculus provides for RNA-Seq applications that segment reads (Oculus can significantly benefit the alignment of many 25mers), but Oculus may also yield benefit to customized bioinformatics analyses that take similar approaches. Also of note is that for highly-sensitive but slow aligners such as BLAT [21] and Smith-Waterman [22], Oculus’s relative runtime will be insignificant (i.e., s_a_/s_o_ - > 0), so streaming read alignment will be of greater use to applications that require such sensitivity. Perhaps most importantly, as sequencing throughput increases so too will read redundancy and the marginal benefit of compressing input reads, though this will be mitigated by longer read lengths and paired-end reads.

To be effective, Oculus requires read redundancy and an aligner that does not already exploit that redundancy. To be consistent, Oculus requires the aligner to ignore quality score and use parameters that guarantee deterministic behavior. By default, Bowtie will report one alignment at random for ambiguously mapping reads, and Oculus by definition cannot produce multiple alignments for a single read sequence. The exception to this is if the aligner is configured to report multiple alignments per read, either on single or multiple SAM lines, in which case Oculus will reconstitute the reads aligning to each location.

Since both Bowtie and BWA use left-end seeds, it makes sense that Oculus may report different alignments for reverse-complemented single-end reads. However, we were surprised to find alignment differences for paired-end reads with reversed order. Read order shouldn’t matter in paired-end alignment: since the read orientation remains the same, so should the seeds. Developers who wish to incorporate streaming read compression into their aligners may be interested in exploring this phenomenon.

Another surprising result was that Oculus + Bowtie actually outperformed compression for the second ChIP-Seq data set (it ran in 27.0% of the original time, on 35% of the original data set). Stranger still, the runtime data for that dataset was not noisy – each of the three tests ran in < 28% of the original time. It is possible that Oculus may have compressed a disproportionately large number of slow-aligning reads – reads that take longer to align to the human genome. Better understanding this phenomenon may be a key to further alignment algorithm improvements.

Though Oculus provides immediate benefit to RNA-Seq alignment, further performance gains may be possible by harnessing the idea of streaming read compression. Although implemented here as a customizable “attachment” to a sequential aligner, the streaming compression algorithm could be integrated directly into alignment kernels. One obvious benefit of this would be the ability to store paired-end reads individually (with an extra bit denoting the read number) thereby leveraging additional redundancy (see Figure 1). A more nuanced logical continuation of this idea would be for aligners to use cache objects that retain in memory the alignments of the mostly commonly occurring reads. If present, a skew toward very common reads away from reads with few copies could create the perfect conditions for caching. The combinatorics of sequence length suggests an even greater benefit in storing and reusing alignments of common seed sequences, either in a complete object or a cache.

There are three limitations of Oculus’s current implementation of streaming read compression: FASTQ quality scores are lost, read names are lost beyond the first instance of the sequence, and the order of the reads in the output will not be consistent with normal aligner output. Quality scores and read names can be restored to the final output at the cost of computation time and memory, which adds value for downstream analyses such as SNP calling. However, the alignment itself is still performed without quality scores, which can alter alignment results. In cases where little faith is placed in the read quality scores this may be acceptable, but to mitigate this loss otherwise, we suggest the use of read filtering or trimming as a preprocessing step.

## Conclusion

Oculus provides a demonstrable speed improvement in aligning redundant data, with high fidelity and low memory cost. Further, streaming read compression of redundant reads is generally useful; aligning the unique set of reads is faster than the full set since the overhead of compression is sufficiently low. We expect streaming read compression will play an important role in RNA-Seq alignment and potentially other sequencing applications in the future as data grows and algorithms improve.

## Availability and requirements

**Project Name**: Oculus

**Project Home Page:**http://code.google.com/p/oculus-bio

**Operating system:** Platform independent

**Programming language:** C++

**Other requirements:** Perl version 5 or higher (for configuration), g++ version 4.1.2 or higher (lower versions may work but are untested), Bowtie or BWA (versions 0.12.7 or 0.5.9-r16, respectively), or another SAM-compatible alignment algorithm

**License:** GNU GPL v3

## Abbreviations

DNA: Deoxyribonucleic acid; BWA: Burroughs-Wheeler aligner; RNA-Seq: Ribonucleic acid sequencing; ChIP-Seq: Chromatin immunoprecipitation sequencing; SAM: Sequence alignment map; MAQ: Mapping and Assembly with Quality; BLAST: Basic local alignment search tool; STL: Standard library; ASCII: American standard code for information interchange; I/O: Input/output; CPU: Central processing unit; GHz/MHz: Gigahertz/Megahertz; DDR3: Double data rate type 3; OS: Operating system; GB/MB: Gigabyte/Megabyte; PCR: Polymerase chain reaction; BLAT: BLAST-like alignment tool; GPL: GNU public license; GNU: GNU’s not Unix.

## Competing interests

The authors declare that they have no competing interests.

## Authors’ contributions

BAV provided the original idea, wrote the algorithm, performed the benchmarking, modeling, and data interpretation, and drafted the manuscript. MKI contributed critical feedback on the manuscript, suggestions for datasets, and the reverse complement idea. AMC contributed critical feedback on the manuscript and the project, and provided the computational resources necessary to carry out the work. All authors read and approved the final manuscript.

## Supplementary Material

Additional file 1 **Table S1.** Oculus performance statistics. Detailed benchmarking data used in generating runtime figures. Click here for file
